# 

**Published:** 1895-05-11

**Authors:** 


**Notices of Births, Deaths, and Marriages** are inserted in this column at a charge of 2s. 6d. for an announcement not exceeding four lines.

MARRIAGE.

WESTON—CHAPMAN.—On the 5th inst., at St. John's Church, Waterloo, James, son of J. P. Weston, of London, to S. J. (Minnie) Chapman, daughter of T. C. Chapman, of Liverpool.

**Notice to Advertisers and Subscribers.**

All Advertisements, Orders for Copies of The Hospital and Business Communications should be addressed to **The Manager, NOT TO THE EDITOR,** The Hospital, 428, Strand, London, W.C.

The Cost of Subscription, post free, is as follows:—Per week, 2½d.; per quarter, 2s. 8d.; per half-year, 5s. 3d.; per annum, 10s. 6d. Foreign:—Per Annum, 15s. 2d.; payable, in all cases, in advance.

**NOTICE TO CORRESPONDENTS.**

All MS., Letters, Books for Review, and other matters intended for the Editor should be addressed The Editor, The Lodge, Porchester Square, London, W.

The Editor cannot undertake to return rejected MS., even when accompanied by stamped directed envelope.

**"Burdett's Hospital Annual," 1895,**

Ready Immediately.

Sixth year of publication. About 1000 pp. Scarlet cloth, gilt lettered. Price 5s. A most complete guide to British, Colonial, Indian, and American Hospitals, Dispensaries, Nursing and Convalescent Institutions, and Asylums. A few copies of the "Annual" for 1894 may still be obtained on application to the Publishers, The Scientific Press (Limited), 428, Strand, London, W.C.

**NOTICE.—Vol. XVII. of "The Hospital" (October, 1894, to April, 1895), in handsome cloth binding, is now on sale at the Office of this Journal. Price 6s. Cases for binding the Numbers contained in this Volume, 1s. 6d. Index to Vol. XVII., 2½d., post free.**

### Scraps and Gleanings.

The Duchess of Albany visited the Royal Hospital for Children and Women, Waterloo Bridge Road, last week.

Baron de Hirsch has sent a donation of £300 to the North-Eastern Hospital for Children, Hackney Road, Shoreditch.

Cholera is increasing its ravages again in Russia with the warm weather. In one province 56 cases were registered, four of which proved fatal.

Over 1,000 people attended the annual ball in aid of the Metropolitan and City Police Orphanage, which took place recently at the Cannon Street Hotel.

At Haslingden, the Health and Sanitary Committee have resolved that a small-pox hospital shall be erected for the locality, accommodation for such cases being imperative.

Princess Henry of Battenberg has promised to open a grand bazaar at St. Martin's Town Hall on the 14th inst. in aid of the building fund of the Royal Westminster Ophthalmic Hospital.

The Princess of Wales has promised to attend the bazaar to be help in June in aid of the National Society for the Prevention of Cruelty to Children. Her Majesty the Queen will be a patroness.

The debt on the Redcliffe Infirmary, Oxford, with which the year commenced, has unfortunately been further increased by £625, making a total indebtedness of £2 626 on the balance-sheet for the year.

Various workshops and collieries scattered throughout Nottinghamshire and Derbyshire have, together with the various friendly societies, contributed £130 to the Nottingham and Midland Eye Infirmary.

The trustees of the Roger Lyon Jones trust have given £250 towards the funds of St. Paul's Eye and Ear Hospital at Liverpool on condition that it is appropriated to the reduction of the existing mortgage of £1,500.

The Ironmongers' Company and the Lion Brewery Company have each contributed 100 guineas, and the Wax Chandlers' Company 25 guineas to the special fund for opening the closed wards of St. Thomas's Hospital.

In Berlin an English Speaking Medical Society has been organised. The society is mainly for English and American medical men, but we believe other nationalities would be admitted to membership if familiar with the English language.

Madame Sacara Tulbure, the brilliant student of medicine in Roumania, is debarred from election to the vacant Professorship of Children's Diseases from the fact that women are debarred from voting in the Senate, where the medical faculty is represented by election.

The Duchess of Albany will attend the annual meeting of the Children's Country Holidays Fund on May 17th, at the Hotel Metropole.

The Emperor of Japan has conferred the third class decoration of the Order of the Rising Sun upon Mr. W. Anderson, surgeon to St. Thomas's Hospital. Mr. Anderson worked for some years in Japan.

The members of The Hospital Dramatic Club performed the farce, "A Regular Fix," at St. Bartholomew's Hospital on the occasion last week of the conversazione in honour of the one hundredth year of its existence.

By command of the Prince of Wales, the Hon. A. Lyttelton has received a donation of £5 out of the funds of the Duchy of Cornwall for the Children's Country Holiday Fund, which is just now in great need of money.

The Marlowe Dramatic Club announce a special performance to be given in St. George's Hall, Langham Place, on Saturday, 18th inst., of the Irish drama "Arrah-na-Pogue," in aid of the funds of the Great Northern Central Hospital, N.

From every point of view the bazaar in aid of the furnishing fund of the Derbyshire Royal Infirmary was a prodigious success. Including donations, the sum realised was £4,300, a far larger sum than has ever before been obtained at any previous undertaking of a similar character in the town and county of Derby.

The appeal made for funds for the Brignorth and South Shropshire Infirmary met with a most liberal response. Mr. and Mrs. Foster, of Apley Park, gave a donation of £1,000, whilst another £7,000 was generously subscribed by a lady in the neighbourhood. The foundation-stone of this building has now been laid.

The Committee of the Coventry and Warwickshire Hospital, in making an appeal for financial support for their institution, assert that unless the income of the hospital is much increased they must adopt one of the following alternative courses: either to close one of the wards, or to again draw on the capital to meet increased expenditure.

It has been decided by the Committee of Management of the Union Vélocipédique de France to appoint a Committee of Medical Assistance, the object of this being, we are told, to try and "minimise the evil consequences of accidents occurring to cyclists, and to study questions belonging to the domain of medicine." Medical stations, fixed and movable, for the treatment of accidents, it is also hoped will be an outcome of such endeavour.

### The Student of Medicine.

### APPOINTMENTS.

Dr. Davern, appointed Medical Officer for the Holborn Union School at Mitcham. J. E. Gemmell, M.B., C.M.Edin., appointed Honorary Hospital Medical Officer to the Ladies' Charity and Lying-in Hospital, Liverpool. T. B. Grimsdale, M.B., M.R.C.S., appointed Honorary Hospital Medical Officer to the Ladies' Charity and Lying-in Hospital, Liverpool. G. J. Hines, L.R.C.S.I., L.M., appointed Medical Officer for the No. 4 District of the Hollingbourn Union. A. M. Hines, M.R.C.S., L.R.C.P., appointed Surgeon to the Chisenhale Street Bridewell, Liverpool. G. O. Jacobson, L.R.C.P.Lond., M.R.C.S.Eng., appointed Medical Officer for the Pembridge District of the Kington Union. Dr. J. J. MacGregor, appointed Assistant Surgeon to the Colchester Hospital. T. Mackenzie, M.A., M.D.Edin., appointed Physician to Noble's Isle of Man General Hospital and Dispensary. Dr. G. H. Marshall, appointed Assistant Medical Officer to the St. Marylebone Infirmary. Dr. R. A. Moss, appointed Medical Officer for the Milton District of the Sculcoates Union. V. Moxey, appointed Honorary Medical Officer Holloway and North Islington Dispensary. B. H. Nicholson, M.B., C.M.Edin., appointed Assistant Surgeon to the Colchester Hospital. Dr. J. O'Donnell, appointed Medical Officer to the Dublin Metropolitan Police Medical Aid Association. H. Payne, M.D., M.R.C.S., L.S.A., appointed Honorary Medical Officer to the Ashton-under-Lyne District Infirmary. Dr. J. B. O. Richards, appointed Medical Officer for the Milton Abbott District of the Tavistock Union. Dr. Solly, appointed Surgeon to the Exeter Dispensary. A. H. Willoughby, L.R.C.P.Lond., M.R.C.S.Eng., appointed Medical Officer of the Sunbury District of the Staines Union. T. B. Young, M.D.Brux., M.R.C.S.Eng., appointed Medical Officer of Health to the Halesowen Rural District Council.

### VACANCIES.

The following vacancies are announced this week, the amount of salary in each case being given after the name of the institution:

#### Resident Medical Officers.

Darlington Hospital and Dispensary, Darlington.—House Surgeon, doubly qualified, registered, and unmarried. Salary £100 per annum, with board and lodging. Applications and testimonials to the Secretaries, 80, Bondgate, Darlington, by May 16th.

West Riding Asylum, Wadsley, near Sheffield.—Fifth Assistant Medical Officer. Salary £100 per annum, rising £10 a year to £150, with board, &c. Applications to the Secretary by May 15th.

Worcester General Infirmary, Worcester.—Assistant House Surgeon and Dispenser, fully qualified; unmarried. Salary £70 per annum, with board, residence, and washing. Office tenable for not more than two years. Applications and testimonials to Secretary, Worcester Chambers, Pierpoint Street, Worcester, by May 18th.

#### Non-Resident Medical Officers.

Borough of Stockton-on-Tees.—Medical Officer of Health and Medical Superintendent for the Fever Hospital. Salary £300 per annum. Applications and testimonials to Mat. B. Dodds, Town Clerk, by May 14th.

### THE STUDENT OF MEDICINE—*continued.*

Denbighshire Infirmary and General Dispensary, Denbigh.—Honorary Medical Officer, doubly qualified. Applications to the Chairman of the Committee of Management by May 14th.

Dental Hospital of London, Leicester Square.—Two Dental Surgeons. Candidates must be Licentiates of Dental Surgery of one of the licensing bodies recognised by the General Medical Council. Applications and testimonials to J. Francis Pink, Secretary, by June 10th.

North-Eastern Hospital for Children, Hackney Road, Shoreditch, N.E.—Physician to the Out-patients; must be Fellows or Members of the Royal College of Physicians of London. Applications to the Secretary by May 14th.

St. Thomas's Hospital Medical School.—Lecturer on Physiology. Applications to the Dean by May 25th.

Westminster General Dispensary, 9, Gerrard Street, Soho, W.—Honorary Surgeon. Applications and testimonials to J. J. Johnson, Secretary, by May 20th.

### UNIVERSITIES AND COLLEGES.

#### UNIVERSITY OF LONDON.

The following examiners have been appointed for 1835 6 :—Medicine : Dr. Cavafy and Dr. Frank Payne. Surgery : Mr. William Anderson and Mr. Henry Morris. Obstetric Medicine : Dr. Herman and Dr. Horrocks. Materia Medica and Pharmaceutical Chemistry : Dr. Sidney Phillips and Dr. Hale White. Forensic Medicine : Dr. Luff and Professor Dixon Mann. Comparative Anatomy : Mr. F. E. Beddard and Professor Ray Lankester. Anatomy : Professor D. J. Cunningham and Mr. Clement Lucas. Physiology : Professor W. D. Halliburton and Professor W. Stirling. Chemistry : Professor Wyndham Dunstan and Professor Herbert McLeod. State Medicine : Dr. Edward Seaton and Dr. Whitelegge. Mental Physiology : Dr. T. Claye Shaw and Professor Sully, LL.D.

#### UNIVERSITY COLLEGE, DUNDEE.

At the last meeting of the Education Board of University College, Dundee, a letter was read from the Edinburgh University Committee stating that that University had resolved to recognise University College, Dundee, as a medical school, the courses of instruction in which qualify for graduation in medicine, pending the re-establishment of the union with the University of St. Andrews. The recognition extends to women students. The Dundee Royal Infirmary has also been recognised as a hospital fulfilling the conditions of Ord. 16, Sec. vii. (5 and 10).

#### UNIVERSITY OF DURHAM.

The following degrees were conferred at a convocation held on April 27th :—

Doctor of Medicine (Practitioners).—W. P. Ashe, E. Ferrand, S. Harris, B. Jones, H. A. Latimer, W. Moxon, J. Taylor, E. S. Smith, T. F. Young.

Doctor of Medicine.—G. H. V. Appleby, F. W. Fullerton, R. Green, W. W. Hodgins, W. H. Maidlow, F. H. Marson, B. B. Thorne-Thorne.

Doctor of Hygiene.—W. H. Turnbull.

Master in Surgery.—W. Martin.

Bachelor of Medicine.—C. A. Brough, J. A. H. White, J. R. Prior, E. Turner, S. H. Hanley, H. D. Senior, N. Bennett, R. M. Le H. Cooper, W. I'A. Charlton, P. Davidson, E. Fielden, F. C. Ford, E. R. Fothergill, E. E. Frazer, J. R. Fuller, R. W. Gilmour, G. Gocher, C. Hanks, E. R. Kendall, W. R. Kingdon, G. E. Middlemist, M. S. Paterson, W. H. Peake, T. Sanderson, E. P. Satchell, E. W. Scott.

Bachelor in Surgery.—N. Bennett, W. I'A. Charlton, R. M. Le H. Cooper, P. Davidson, E. Fielden, E. R. Fothergill, E. E. Frazer, J. R. Fuller, R. W. Gilmour, G. Gocher, C. Hanks, M. S. Paterson, W. H. Peake, J. R. Prior, T. Sanderson, E. P. Satchell, G. W. Scott, E. Turner, J. A. H. White.

Bachelor of Hygiene.—G. J. Awburn, G. H. V. Appleby, E. Turner, E. C. Willcox.

Bachelor of Surgery.—R. Beattie.

Diploma in Public Health.—W. H. Symons.

